# [1,2-Bis(diphenyl­phosphan­yl)ethane-2κ^2^ *P*,*P*′]tetra­carbonyl-1κ^3^ *C*,2κ*C*-(μ-2-cyclo­pentyl-2-aza­propane-1,3-dithiol­ato-1:2κ^4^ *S*,*S*′:*S*,*S*′)diiron(II)(*Fe*—*Fe*)

**DOI:** 10.1107/S1600536811040347

**Published:** 2011-10-08

**Authors:** Youtao Si, Hui Chen, ChangNeng Chen

**Affiliations:** a State Key Laboratory Breeding Base of Humid Subtropical Mountain Ecology, College of Geographical Sciences, Fujian Normal University, Fuzhou 350007, People’s Republic of China; b State Key Laboratory of Structure Chemistry, Fujian Institute of Research on the Structure of Matter, Fuzhou, Fujian 350002, People’s Republic of China

## Abstract

In the title compound, [Fe_2_(C_7_H_13_NS_2_)(C_26_H_24_P_2_)(CO)_4_], the Fe_2_S_2_ core exhibits a butterfly-like shape, with two S atoms bridging the Fe–Fe dumbbell. Each of the two Fe atoms exhibits a distorted octa­hedral environment. One Fe atom is additionally bonded to three carbonyl C atoms, whereas the other Fe atom is additionally bonded to one carbonyl C atom and two P atoms of the chelating dppe [dppe = 1,2-bis­(diphenyl­phosphan­yl)ethane] ligand. Non-classical intra­molecular C—H⋯S hydrogen-bonding inter­actions are present in the structure. The packing of adjacent mol­ecules along [100] is accomplished mainly through van der Waals forces.

## Related literature

For background to Fe-only hydrogenases, see: Darensbourg *et al.* (2000 ▶); Lawrence *et al.* (2001 ▶). For synthetic details, see: Li & Rauchfuss (2002 ▶). 
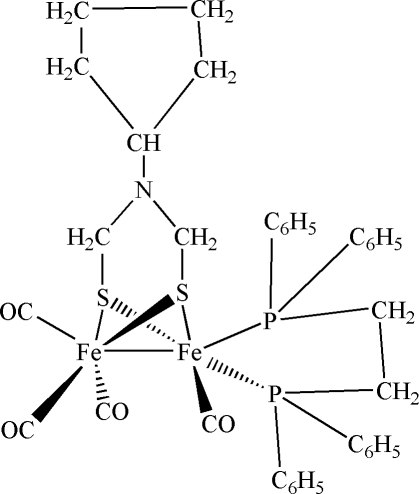


## Experimental

### 

#### Crystal data

[Fe_2_(C_7_H_13_NS_2_)(C_26_H_24_P_2_)(CO)_4_]
*M* *_r_* = 797.44
Triclinic, 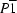

*a* = 11.763 (10) Å
*b* = 12.402 (12) Å
*c* = 13.284 (13) Å
α = 84.66 (3)°
β = 78.19 (3)°
γ = 76.33 (3)°
*V* = 1841 (3) Å^3^
*Z* = 2
Mo *K*α radiation
μ = 1.03 mm^−1^
*T* = 294 K
0.12 × 0.09 × 0.08 mm

#### Data collection

Oxford Diffraction Xcalibur diffractometer
Absorption correction: multi-scan (*CrystalClear*; Rigaku, 2005 ▶) *T*_min_ = 0.840, *T*_max_ = 1.000
13988 measured reflections
8063 independent reflections
4461 reflections with *I* > 2σ(*I*)
*R*_int_ = 0.046

#### Refinement

*R*[*F* ^2^ > 2σ(*F* ^2^)] = 0.069
*wR*(*F* ^2^) = 0.209
*S* = 1.07
8058 reflections
433 parameters
H-atom parameters constrained
Δρ_max_ = 0.73 e Å^−3^
Δρ_min_ = −0.66 e Å^−3^

### 

Data collection: *CrystalClear* (Rigaku, 2005 ▶); cell refinement: *CrystalClear*; data reduction: *CrystalClear*; program(s) used to solve structure: *SHELXS97* (Sheldrick, 2008 ▶); program(s) used to refine structure: *SHELXL97* (Sheldrick, 2008 ▶); molecular graphics: *WinGX* (Farrugia, 1999 ▶); software used to prepare material for publication: *publCIF* (Westrip, 2010 ▶).

## Supplementary Material

Crystal structure: contains datablock(s) I, global. DOI: 10.1107/S1600536811040347/wm2535sup1.cif

Structure factors: contains datablock(s) I. DOI: 10.1107/S1600536811040347/wm2535Isup2.hkl

Additional supplementary materials:  crystallographic information; 3D view; checkCIF report

## Figures and Tables

**Table 1:** Selected bond lengths (Å)

Fe2—C4	1.766 (7)
Fe2—P1	2.224 (2)
Fe2—P2	2.265 (2)
Fe2—S1	2.282 (2)
Fe2—S2	2.289 (2)
Fe2—Fe1	2.583 (2)
Fe1—C3	1.786 (6)
Fe1—C1	1.791 (7)
Fe1—C2	1.814 (7)
Fe1—S1	2.281 (2)
Fe1—S2	2.286 (2)

**Table 2:** Hydrogen-bond geometry (Å, °)

*D*—H⋯*A*	*D*—H	H⋯*A*	*D*⋯*A*	*D*—H⋯*A*
C21—H21⋯S1	0.93	2.77	3.490 (7)	135
C27—H27⋯S1	0.93	2.84	3.416 (7)	122

**Fig. 1.**
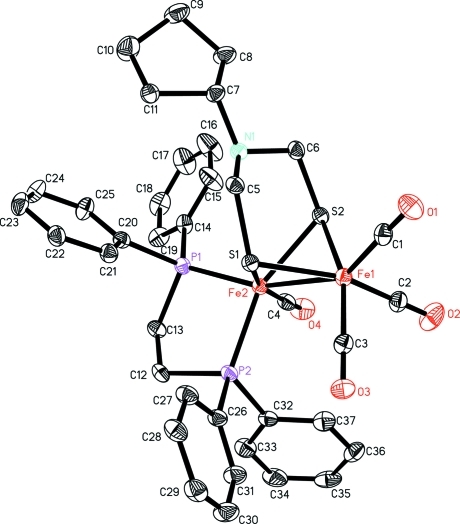
The molecular structure of the title compound, with atom labels and 20% probability displacement ellipsoids for all non-H atoms.

**Fig. 2.**
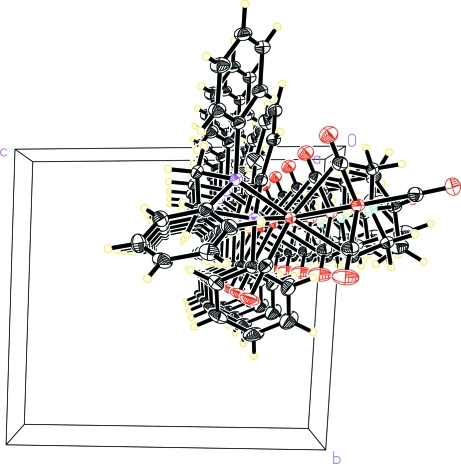
The packing diagram of the title compound, viewed down the a axis.

## supplementary crystallographic information

### Comment

Hydrogenases are capable of efficiently catalysing the oxidation of molecular hydrogen or its production from protons and electrons (Darensbourg *et al.*, 2000; Lawrence *et al.*, 2001). The well-known active sites of intensively studied Fe—Fe hydrogenases include Fe_2_S_2_ clusters and a cuboidal Fe_4_S_4_ unit, with the former playing an important role in the catalytic process.

The title compound is a mimic of the Fe_2_S_2_ cluster. As shown in Fig. 1, the two Fe atoms are linked through an Fe—Fe single bond and further bridged by two S atoms. Thus a butterfly arrangement is formed, with the dihedral angle between the two Fe_2_S planes being 73.28 (8)° and the average Fe—S bond length 2.285 Å. Each Fe atom exhibits a distorted octahedral environment. Atom Fe1 is bonded to three carbonyl C atoms, whereas atom Fe2 is bonded to one carbonyl C atom and two P atoms of the chelating dppe [dppe = 1,2-bis(diphenylphosphanyl)ethane] ligand. Notably, the P atoms of dppe have substituted two carbonyl C atoms at Fe2, with one P atom in apical position and the other in basal position. Because the Fe–Fe dumbbell is asymetrically substituted, there is an obvious difference among Fe—C bond lengths (Table 1; average value 1.79 Å). The Fe2—P2 bond is 0.04 Å longer than the Fe2—P1 bond due to steric effects, and the average Fe—P bond length is 2.25 Å. The P—Fe—P angle [88.10 (7)°] is much smaller than the mean C—Fe—C bond angle [97 (5)°], because of the rigidity of the dppe ligand. Non-classical intramolecular C—H···S hydrogen bonding interactions (Table 2) are present in the structure.

The packing diagram is shown in Fig. 2. The packing of adjacent molecules along [100] is accomplished mainly through van der Waals forces.

### Experimental

The synthesis of the title compound was carried out under an dry, purified, oxygen-free nitrogen atmosphere using standard Schlenk techniques. Solvents, such as THF and hexane, were dried according to standard methods. Commercially available products, like paraformaldehyde, [Fe(CO)_5_], LiBEt_3_H, F_3_CCOOH, dppe and C_5_H_9_NH_2_ were of reagent grade and used as received. The starting material [Fe_2_(SH)_2_(CO)_6_] was prepared as documented. The title compound was prepared by a condensation of Fe_2_(SH)_2_(CO)_6_ with formaldehyde in the presence of cyclopentyamie (Li & Rauchfuss, 2002), followed by substitution of carbonyls by dppe (1,2-bis(diphenylphosphanyl)ethane).

[Fe_2_S_2_(CO)_6_] (1 mmol, 0.344 g) was dissolved in dry THF (40 ml) under a nitrogen atmosphere and then cooled to 195 K with acetone and liquid nitrogen. After the solution was stirred for 30 minutes, LiBEt_3_H (2 mmol) was added dropwise very slowly. At the midpoint of the addition, the color of the reaction mixture turned from red to dark green; for the rest of addition it remained green. After another 30 minutes, F_3_CCOOH (2 mmol, 0.149 ml) was added. The new mixture was stirred for an additional hour. The cool solution was added to a mixture of paraformaldehyde (40 mmol, 1.2 g) and C_5_H_9_NH_2_ (1 mmol) in THF which had been stirred for 10 h and cooled to 273 K. The last mixture was stirred for 24 h and the majority of the solvent was evaporated under vacuum. The remaining residual was filtered through silica gel. A red fraction was collected by elution with hexane. 1 mmol (excess) dppe was added to the red fraction, and the solution gradually became purple, after which the solution was stirred for another 3 h. Recrystallization of the crude purple product from freshly distilled pentane in a refridgerator at 253 K for several days produced crystals in moderate yield (~60%) suitable for X-ray crystallography.

### Refinement

Hydrogen atoms were placed at idealized positions and allowed to ride on their parent atoms, with CH_2_ and CH_3_ bonds set equal to 0.97 and 0.96 Å, respectively. For all H atoms, *U*_iso_(H) = 1.2*U*_eq_(C). The highest residual peak was located at 0.06 Å from Fe2. Reflections 111, 110, 011, 011, and 101 were affected by the beam stop and were omitted from the refinement.

### Crystal data

**Table:**

[Fe_2_(C_7_H_13_NS_2_)(C_26_H_24_P_2_)(CO)_4_]	*Z* = 2
*M**_r_* = 797.44	*F*(000) = 824
Triclinic, *P*1	*D*_x_ = 1.438 Mg m^−^^3^
Hall symbol: -P 1	Mo *K*α radiation, λ = 0.71073 Å
*a* = 11.763 (10) Å	Cell parameters from 3975 reflections
*b* = 12.402 (12) Å	θ = 2.7–27.2°
*c* = 13.284 (13) Å	µ = 1.03 mm^−^^1^
α = 84.66 (3)°	*T* = 294 K
β = 78.19 (3)°	Prism, dark red
γ = 76.33 (3)°	0.12 × 0.09 × 0.08 mm
*V* = 1841 (3) Å^3^	

### Data collection

**Table:**

Oxford Diffraction Xcalibur diffractometer	8063 independent reflections
Radiation source: fine-focus sealed tube	4461 reflections with *I* > 2σ(*I*)
graphite	*R*_int_ = 0.046
Detector resolution: 28.5714 pixels mm^-1^	θ_max_ = 27.2°, θ_min_ = 2.2°
ω and φ scans	*h* = −14→15
Absorption correction: multi-scan (*CrystalClear*; Rigaku, 2005)	*k* = −13→15
*T*_min_ = 0.840, *T*_max_ = 1.000	*l* = −17→17
13988 measured reflections	

### Refinement

**Table:**

Refinement on *F*^2^	Primary atom site location: structure-invariant direct methods
Least-squares matrix: full	Secondary atom site location: difference Fourier map
*R*[*F*^2^ > 2σ(*F*^2^)] = 0.069	Hydrogen site location: inferred from neighbouring sites
*wR*(*F*^2^) = 0.209	H-atom parameters constrained
*S* = 1.07	*w* = 1/[σ^2^(*F*_o_^2^) + (0.0941*P*)^2^ + 0.4758*P*] where *P* = (*F*_o_^2^ + 2*F*_c_^2^)/3
8058 reflections	(Δ/σ)_max_ < 0.001
433 parameters	Δρ_max_ = 0.73 e Å^−^^3^
0 restraints	Δρ_min_ = −0.66 e Å^−^^3^

### Special details

**Table:**

Geometry. All esds (except the esd in the dihedral angle between two l.s. planes) are estimated using the full covariance matrix. The cell esds are taken into account individually in the estimation of esds in distances, angles and torsion angles; correlations between esds in cell parameters are only used when they are defined by crystal symmetry. An approximate (isotropic) treatment of cell esds is used for estimating esds involving l.s. planes.
Refinement. Refinement of F^2^ against ALL reflections. The weighted R-factor wR and goodness of fit S are based on F^2^, conventional R-factors R are based on F, with F set to zero for negative F^2^. The threshold expression of F^2^ > 2sigma(F^2^) is used only for calculating R-factors(gt) etc. and is not relevant to the choice of reflections for refinement. R-factors based on F^2^ are statistically about twice as large as those based on F, and R- factors based on ALL data will be even larger.

### Fractional atomic coordinates and isotropic or equivalent isotropic displacement parameters (Å^2^)

**Table:**

	*x*	*y*	*z*	*U*_iso_*/*U*_eq_	
Fe2	0.41292 (7)	0.25705 (6)	0.22097 (6)	0.0369 (2)	
Fe1	0.31542 (7)	0.22815 (6)	0.07103 (6)	0.0414 (2)	
S2	0.42334 (13)	0.35974 (11)	0.06835 (11)	0.0410 (3)	
P1	0.56027 (13)	0.25745 (12)	0.30241 (11)	0.0408 (4)	
S1	0.48107 (12)	0.11162 (10)	0.11497 (11)	0.0383 (3)	
P2	0.33243 (14)	0.16019 (12)	0.35931 (12)	0.0424 (4)	
N1	0.6532 (4)	0.2314 (4)	0.0138 (4)	0.0418 (11)	
C5	0.6158 (5)	0.1258 (5)	0.0199 (4)	0.0431 (13)	
H5A	0.6814	0.0667	0.0350	0.052*	
H5B	0.6029	0.1138	−0.0476	0.052*	
C21	0.7043 (5)	0.0418 (5)	0.2622 (5)	0.0475 (14)	
H21	0.6363	0.0248	0.2485	0.057*	
C7	0.7746 (5)	0.2213 (5)	−0.0525 (5)	0.0469 (14)	
H7	0.7727	0.1926	−0.1183	0.056*	
C26	0.3043 (5)	0.0186 (5)	0.3614 (5)	0.0466 (14)	
C20	0.7012 (5)	0.1498 (5)	0.2865 (4)	0.0440 (13)	
C11	0.8735 (5)	0.1388 (5)	−0.0039 (5)	0.0529 (15)	
H11A	0.8445	0.1221	0.0683	0.063*	
H11B	0.8999	0.0700	−0.0397	0.063*	
C29	0.2679 (7)	−0.1986 (5)	0.3619 (5)	0.0613 (18)	
H29	0.2555	−0.2699	0.3615	0.074*	
C2	0.1821 (6)	0.3386 (5)	0.0909 (5)	0.0552 (16)	
C27	0.3942 (6)	−0.0665 (5)	0.3120 (5)	0.0562 (16)	
H27	0.4668	−0.0513	0.2789	0.067*	
C28	0.3753 (7)	−0.1745 (5)	0.3122 (5)	0.0587 (17)	
H28	0.4351	−0.2299	0.2788	0.070*	
C32	0.1888 (5)	0.2396 (5)	0.4284 (5)	0.0476 (14)	
C25	0.8064 (5)	0.1740 (5)	0.3036 (5)	0.0561 (17)	
H25	0.8068	0.2456	0.3188	0.067*	
C6	0.5649 (5)	0.3214 (5)	−0.0233 (5)	0.0461 (14)	
H6A	0.5484	0.2997	−0.0862	0.055*	
H6B	0.5988	0.3864	−0.0403	0.055*	
O1	0.3405 (5)	0.1815 (4)	−0.1468 (4)	0.0742 (14)	
O3	0.1806 (4)	0.0572 (4)	0.1566 (4)	0.0658 (13)	
C14	0.6148 (6)	0.3867 (5)	0.2996 (5)	0.0493 (15)	
C22	0.8095 (6)	−0.0418 (5)	0.2583 (5)	0.0522 (15)	
H22	0.8096	−0.1139	0.2442	0.063*	
O2	0.0965 (5)	0.4090 (4)	0.1033 (5)	0.0948 (19)	
C23	0.9120 (6)	−0.0174 (6)	0.2752 (5)	0.0614 (18)	
H23	0.9818	−0.0723	0.2716	0.074*	
C8	0.8162 (6)	0.3313 (6)	−0.0757 (6)	0.0657 (19)	
H8A	0.7885	0.3784	−0.0173	0.079*	
H8B	0.7883	0.3714	−0.1354	0.079*	
C24	0.9100 (6)	0.0909 (6)	0.2979 (6)	0.067 (2)	
H24	0.9791	0.1078	0.3093	0.080*	
C1	0.3323 (6)	0.2011 (5)	−0.0619 (5)	0.0511 (15)	
C3	0.2328 (5)	0.1256 (5)	0.1234 (5)	0.0490 (14)	
C12	0.4347 (6)	0.1366 (5)	0.4513 (4)	0.0496 (14)	
H12A	0.3902	0.1306	0.5208	0.060*	
H12B	0.4930	0.0673	0.4376	0.060*	
C19	0.6330 (6)	0.4289 (6)	0.3881 (6)	0.0623 (18)	
H19	0.6125	0.3951	0.4527	0.075*	
C33	0.1659 (7)	0.2628 (6)	0.5333 (5)	0.0650 (18)	
H33	0.2251	0.2353	0.5718	0.078*	
C18	0.6826 (7)	0.5228 (6)	0.3780 (7)	0.073 (2)	
H18	0.6956	0.5509	0.4361	0.088*	
C31	0.1982 (6)	−0.0077 (5)	0.4136 (5)	0.0587 (17)	
H31	0.1393	0.0465	0.4497	0.070*	
C37	0.0973 (6)	0.2850 (5)	0.3732 (6)	0.0613 (18)	
H37	0.1107	0.2726	0.3034	0.074*	
C13	0.4998 (6)	0.2336 (5)	0.4421 (4)	0.0521 (15)	
H13A	0.5640	0.2144	0.4807	0.062*	
H13B	0.4446	0.3002	0.4693	0.062*	
C15	0.6431 (8)	0.4408 (7)	0.2059 (6)	0.084 (3)	
H15	0.6282	0.4156	0.1472	0.100*	
C30	0.1788 (7)	−0.1157 (6)	0.4124 (6)	0.068 (2)	
H30	0.1064	−0.1314	0.4455	0.082*	
C10	0.9750 (7)	0.1999 (7)	−0.0165 (7)	0.085 (3)	
H10A	0.9757	0.2298	0.0482	0.103*	
H10B	1.0512	0.1495	−0.0384	0.103*	
C9	0.9529 (6)	0.2921 (6)	−0.0967 (7)	0.080 (2)	
H9A	0.9824	0.2652	−0.1655	0.096*	
H9B	0.9905	0.3517	−0.0886	0.096*	
C17	0.7117 (9)	0.5727 (6)	0.2841 (8)	0.095 (3)	
H17	0.7444	0.6349	0.2783	0.114*	
C36	−0.0121 (6)	0.3474 (6)	0.4187 (6)	0.0654 (19)	
H36	−0.0720	0.3748	0.3808	0.078*	
C34	0.0576 (8)	0.3254 (7)	0.5809 (6)	0.078 (2)	
H34	0.0442	0.3386	0.6506	0.094*	
C35	−0.0303 (7)	0.3683 (6)	0.5238 (6)	0.075 (2)	
H35	−0.1025	0.4114	0.5553	0.090*	
C16	0.6934 (10)	0.5323 (7)	0.1977 (7)	0.107 (4)	
H16	0.7148	0.5663	0.1335	0.129*	
O4	0.2806 (5)	0.4676 (4)	0.3127 (4)	0.0747 (15)	
C4	0.3300 (6)	0.3821 (5)	0.2772 (5)	0.0524 (15)	

### Atomic displacement parameters (Å^2^)

**Table:**

	*U*^11^	*U*^22^	*U*^33^	*U*^12^	*U*^13^	*U*^23^
Fe2	0.0411 (5)	0.0303 (4)	0.0380 (4)	−0.0066 (3)	−0.0071 (3)	0.0003 (3)
Fe1	0.0435 (5)	0.0357 (4)	0.0463 (5)	−0.0090 (4)	−0.0116 (4)	−0.0004 (3)
S2	0.0464 (8)	0.0298 (7)	0.0456 (8)	−0.0067 (6)	−0.0100 (6)	0.0026 (5)
P1	0.0440 (8)	0.0373 (8)	0.0408 (8)	−0.0066 (6)	−0.0102 (7)	−0.0018 (6)
S1	0.0417 (8)	0.0277 (6)	0.0442 (8)	−0.0068 (6)	−0.0070 (6)	0.0004 (5)
P2	0.0471 (9)	0.0359 (8)	0.0416 (8)	−0.0080 (7)	−0.0061 (7)	0.0024 (6)
N1	0.043 (3)	0.034 (2)	0.048 (3)	−0.011 (2)	−0.007 (2)	0.005 (2)
C5	0.048 (3)	0.037 (3)	0.042 (3)	−0.009 (3)	−0.007 (3)	0.001 (2)
C21	0.051 (4)	0.039 (3)	0.053 (4)	−0.009 (3)	−0.012 (3)	0.000 (3)
C7	0.039 (3)	0.039 (3)	0.056 (4)	−0.005 (3)	−0.001 (3)	−0.001 (3)
C26	0.049 (3)	0.038 (3)	0.049 (3)	−0.011 (3)	−0.002 (3)	0.003 (2)
C20	0.047 (3)	0.046 (3)	0.039 (3)	−0.011 (3)	−0.007 (3)	0.001 (2)
C11	0.050 (4)	0.044 (3)	0.059 (4)	−0.006 (3)	−0.008 (3)	0.007 (3)
C29	0.080 (5)	0.041 (4)	0.071 (5)	−0.025 (4)	−0.022 (4)	0.005 (3)
C2	0.050 (4)	0.049 (4)	0.068 (4)	−0.009 (3)	−0.019 (3)	0.001 (3)
C27	0.065 (4)	0.041 (3)	0.057 (4)	−0.012 (3)	−0.002 (3)	0.003 (3)
C28	0.079 (5)	0.039 (3)	0.054 (4)	−0.009 (3)	−0.008 (4)	0.000 (3)
C32	0.050 (4)	0.042 (3)	0.048 (3)	−0.014 (3)	0.002 (3)	0.001 (3)
C25	0.047 (4)	0.050 (4)	0.076 (5)	−0.006 (3)	−0.021 (3)	−0.019 (3)
C6	0.049 (3)	0.037 (3)	0.052 (4)	−0.009 (3)	−0.015 (3)	0.007 (2)
O1	0.096 (4)	0.069 (3)	0.058 (3)	−0.019 (3)	−0.016 (3)	−0.007 (2)
O3	0.064 (3)	0.065 (3)	0.074 (3)	−0.030 (3)	−0.009 (2)	0.005 (2)
C14	0.058 (4)	0.038 (3)	0.056 (4)	−0.009 (3)	−0.022 (3)	−0.004 (3)
C22	0.054 (4)	0.045 (3)	0.052 (4)	0.000 (3)	−0.012 (3)	0.000 (3)
O2	0.061 (3)	0.055 (3)	0.158 (6)	0.010 (3)	−0.021 (4)	−0.012 (3)
C23	0.047 (4)	0.062 (4)	0.067 (4)	0.010 (3)	−0.014 (3)	−0.011 (3)
C8	0.052 (4)	0.053 (4)	0.084 (5)	−0.017 (3)	0.005 (4)	0.010 (3)
C24	0.037 (4)	0.075 (5)	0.088 (5)	−0.003 (3)	−0.013 (3)	−0.020 (4)
C1	0.052 (4)	0.048 (4)	0.058 (4)	−0.010 (3)	−0.020 (3)	−0.004 (3)
C3	0.047 (3)	0.046 (3)	0.056 (4)	−0.010 (3)	−0.012 (3)	−0.005 (3)
C12	0.064 (4)	0.044 (3)	0.037 (3)	−0.006 (3)	−0.011 (3)	0.006 (2)
C19	0.062 (4)	0.059 (4)	0.067 (5)	−0.014 (3)	−0.006 (3)	−0.020 (3)
C33	0.070 (5)	0.057 (4)	0.058 (4)	−0.006 (4)	0.002 (4)	−0.005 (3)
C18	0.077 (5)	0.054 (4)	0.098 (6)	−0.011 (4)	−0.025 (5)	−0.033 (4)
C31	0.060 (4)	0.051 (4)	0.067 (4)	−0.021 (3)	−0.005 (3)	−0.001 (3)
C37	0.061 (4)	0.055 (4)	0.063 (4)	−0.008 (3)	0.001 (3)	−0.014 (3)
C13	0.062 (4)	0.055 (4)	0.039 (3)	−0.016 (3)	−0.007 (3)	0.000 (3)
C15	0.141 (8)	0.077 (5)	0.061 (5)	−0.063 (6)	−0.045 (5)	0.014 (4)
C30	0.064 (5)	0.054 (4)	0.088 (5)	−0.028 (4)	−0.004 (4)	0.003 (4)
C10	0.067 (5)	0.079 (6)	0.120 (7)	−0.034 (4)	−0.029 (5)	0.016 (5)
C9	0.054 (4)	0.065 (5)	0.110 (7)	−0.019 (4)	0.010 (4)	0.007 (4)
C17	0.143 (9)	0.048 (4)	0.122 (8)	−0.049 (5)	−0.061 (7)	0.013 (5)
C36	0.044 (4)	0.060 (4)	0.088 (6)	−0.008 (3)	−0.006 (4)	−0.009 (4)
C34	0.086 (6)	0.079 (6)	0.052 (4)	−0.012 (5)	0.022 (4)	−0.008 (4)
C35	0.070 (5)	0.051 (4)	0.089 (6)	−0.017 (4)	0.029 (4)	−0.017 (4)
C16	0.189 (11)	0.083 (6)	0.095 (7)	−0.090 (7)	−0.075 (7)	0.027 (5)
O4	0.085 (4)	0.035 (2)	0.087 (4)	0.004 (2)	0.012 (3)	−0.021 (2)
C4	0.060 (4)	0.047 (4)	0.048 (4)	−0.015 (3)	−0.008 (3)	0.009 (3)

### Geometric parameters (Å, °)

**Table:**

Fe2—C4	1.766 (7)	C6—H6A	0.9700
Fe2—P1	2.224 (2)	C6—H6B	0.9700
Fe2—P2	2.265 (2)	O1—C1	1.155 (7)
Fe2—S1	2.282 (2)	O3—C3	1.165 (7)
Fe2—S2	2.289 (2)	C14—C15	1.375 (9)
Fe2—Fe1	2.583 (2)	C14—C19	1.405 (8)
Fe1—C3	1.786 (6)	C22—C23	1.376 (9)
Fe1—C1	1.791 (7)	C22—H22	0.9300
Fe1—C2	1.814 (7)	C23—C24	1.398 (9)
Fe1—S1	2.281 (2)	C23—H23	0.9300
Fe1—S2	2.286 (2)	C8—C9	1.540 (9)
S2—C6	1.840 (6)	C8—H8A	0.9700
P1—C20	1.854 (6)	C8—H8B	0.9700
P1—C14	1.857 (6)	C24—H24	0.9300
P1—C13	1.869 (6)	C12—C13	1.555 (8)
S1—C5	1.847 (6)	C12—H12A	0.9700
P2—C12	1.842 (6)	C12—H12B	0.9700
P2—C32	1.848 (6)	C19—C18	1.405 (9)
P2—C26	1.859 (6)	C19—H19	0.9300
N1—C6	1.458 (7)	C33—C34	1.385 (10)
N1—C5	1.467 (7)	C33—H33	0.9300
N1—C7	1.501 (7)	C18—C17	1.355 (12)
C5—H5A	0.9700	C18—H18	0.9300
C5—H5B	0.9700	C31—C30	1.413 (9)
C21—C20	1.398 (8)	C31—H31	0.9300
C21—C22	1.409 (8)	C37—C36	1.385 (9)
C21—H21	0.9300	C37—H37	0.9300
C7—C8	1.540 (8)	C13—H13A	0.9700
C7—C11	1.562 (8)	C13—H13B	0.9700
C7—H7	0.9800	C15—C16	1.385 (10)
C26—C31	1.394 (8)	C15—H15	0.9300
C26—C27	1.408 (8)	C30—H30	0.9300
C20—C25	1.406 (8)	C10—C9	1.502 (11)
C11—C10	1.533 (9)	C10—H10A	0.9700
C11—H11A	0.9700	C10—H10B	0.9700
C11—H11B	0.9700	C9—H9A	0.9700
C29—C28	1.386 (9)	C9—H9B	0.9700
C29—C30	1.392 (10)	C17—C16	1.368 (11)
C29—H29	0.9300	C17—H17	0.9300
C2—O2	1.160 (8)	C36—C35	1.407 (10)
C27—C28	1.408 (8)	C36—H36	0.9300
C27—H27	0.9300	C34—C35	1.383 (11)
C28—H28	0.9300	C34—H34	0.9300
C32—C37	1.405 (9)	C35—H35	0.9300
C32—C33	1.408 (9)	C16—H16	0.9300
C25—C24	1.390 (9)	O4—C4	1.172 (7)
C25—H25	0.9300		
			
C4—Fe2—P1	90.5 (2)	C24—C25—C20	120.1 (6)
C4—Fe2—P2	89.5 (2)	C24—C25—H25	120.0
P1—Fe2—P2	88.06 (9)	C20—C25—H25	120.0
C4—Fe2—S1	162.7 (2)	N1—C6—S2	114.6 (4)
P1—Fe2—S1	105.84 (8)	N1—C6—H6A	108.6
P2—Fe2—S1	96.46 (9)	S2—C6—H6A	108.6
C4—Fe2—S2	85.6 (2)	N1—C6—H6B	108.6
P1—Fe2—S2	113.34 (8)	S2—C6—H6B	108.6
P2—Fe2—S2	158.03 (7)	H6A—C6—H6B	107.6
S1—Fe2—S2	82.84 (9)	C15—C14—C19	118.7 (6)
C4—Fe2—Fe1	107.3 (2)	C15—C14—P1	118.3 (5)
P1—Fe2—Fe1	157.03 (6)	C19—C14—P1	123.0 (5)
P2—Fe2—Fe1	106.10 (8)	C23—C22—C21	120.4 (6)
S1—Fe2—Fe1	55.51 (6)	C23—C22—H22	119.8
S2—Fe2—Fe1	55.57 (7)	C21—C22—H22	119.8
C3—Fe1—C1	97.4 (3)	C22—C23—C24	119.1 (6)
C3—Fe1—C2	91.7 (3)	C22—C23—H23	120.4
C1—Fe1—C2	102.5 (3)	C24—C23—H23	120.4
C3—Fe1—S1	88.7 (2)	C7—C8—C9	102.7 (5)
C1—Fe1—S1	101.9 (2)	C7—C8—H8A	111.2
C2—Fe1—S1	155.3 (2)	C9—C8—H8A	111.2
C3—Fe1—S2	158.3 (2)	C7—C8—H8B	111.2
C1—Fe1—S2	103.9 (2)	C9—C8—H8B	111.2
C2—Fe1—S2	87.8 (2)	H8A—C8—H8B	109.1
S1—Fe1—S2	82.92 (9)	C25—C24—C23	121.2 (6)
C3—Fe1—Fe2	103.2 (2)	C25—C24—H24	119.4
C1—Fe1—Fe2	148.4 (2)	C23—C24—H24	119.4
C2—Fe1—Fe2	100.5 (2)	O1—C1—Fe1	177.8 (6)
S1—Fe1—Fe2	55.53 (6)	O3—C3—Fe1	178.7 (6)
S2—Fe1—Fe2	55.67 (5)	C13—C12—P2	110.7 (4)
C6—S2—Fe1	109.0 (2)	C13—C12—H12A	109.5
C6—S2—Fe2	114.97 (19)	P2—C12—H12A	109.5
Fe1—S2—Fe2	68.76 (6)	C13—C12—H12B	109.5
C20—P1—C14	101.9 (3)	P2—C12—H12B	109.5
C20—P1—C13	100.5 (3)	H12A—C12—H12B	108.1
C14—P1—C13	103.1 (3)	C14—C19—C18	119.2 (7)
C20—P1—Fe2	123.06 (19)	C14—C19—H19	120.4
C14—P1—Fe2	119.4 (2)	C18—C19—H19	120.4
C13—P1—Fe2	105.7 (2)	C34—C33—C32	122.0 (7)
C5—S1—Fe1	110.9 (2)	C34—C33—H33	119.0
C5—S1—Fe2	112.53 (19)	C32—C33—H33	119.0
Fe1—S1—Fe2	68.96 (7)	C17—C18—C19	120.5 (7)
C12—P2—C32	105.5 (3)	C17—C18—H18	119.7
C12—P2—C26	100.2 (3)	C19—C18—H18	119.7
C32—P2—C26	102.5 (3)	C26—C31—C30	120.8 (6)
C12—P2—Fe2	106.9 (2)	C26—C31—H31	119.6
C32—P2—Fe2	112.7 (2)	C30—C31—H31	119.6
C26—P2—Fe2	126.9 (2)	C36—C37—C32	122.6 (7)
C6—N1—C5	110.3 (4)	C36—C37—H37	118.7
C6—N1—C7	112.1 (4)	C32—C37—H37	118.7
C5—N1—C7	110.1 (4)	C12—C13—P1	107.3 (4)
N1—C5—S1	117.3 (4)	C12—C13—H13A	110.3
N1—C5—H5A	108.0	P1—C13—H13A	110.3
S1—C5—H5A	108.0	C12—C13—H13B	110.3
N1—C5—H5B	108.0	P1—C13—H13B	110.3
S1—C5—H5B	108.0	H13A—C13—H13B	108.5
H5A—C5—H5B	107.2	C14—C15—C16	121.0 (7)
C20—C21—C22	120.7 (6)	C14—C15—H15	119.5
C20—C21—H21	119.6	C16—C15—H15	119.5
C22—C21—H21	119.6	C29—C30—C31	120.1 (6)
N1—C7—C8	114.5 (5)	C29—C30—H30	120.0
N1—C7—C11	112.5 (5)	C31—C30—H30	120.0
C8—C7—C11	106.1 (5)	C9—C10—C11	106.8 (6)
N1—C7—H7	107.8	C9—C10—H10A	110.4
C8—C7—H7	107.8	C11—C10—H10A	110.4
C11—C7—H7	107.8	C9—C10—H10B	110.4
C31—C26—C27	118.4 (6)	C11—C10—H10B	110.4
C31—C26—P2	122.2 (5)	H10A—C10—H10B	108.6
C27—C26—P2	119.4 (5)	C10—C9—C8	103.3 (6)
C21—C20—C25	118.4 (6)	C10—C9—H9A	111.1
C21—C20—P1	121.1 (4)	C8—C9—H9A	111.1
C25—C20—P1	120.4 (4)	C10—C9—H9B	111.1
C10—C11—C7	104.6 (5)	C8—C9—H9B	111.1
C10—C11—H11A	110.8	H9A—C9—H9B	109.1
C7—C11—H11A	110.8	C18—C17—C16	120.5 (7)
C10—C11—H11B	110.8	C18—C17—H17	119.7
C7—C11—H11B	110.8	C16—C17—H17	119.7
H11A—C11—H11B	108.9	C37—C36—C35	118.2 (7)
C28—C29—C30	119.7 (6)	C37—C36—H36	120.9
C28—C29—H29	120.1	C35—C36—H36	120.9
C30—C29—H29	120.1	C35—C34—C33	119.4 (7)
O2—C2—Fe1	179.6 (7)	C35—C34—H34	120.3
C26—C27—C28	120.6 (6)	C33—C34—H34	120.3
C26—C27—H27	119.7	C34—C35—C36	121.0 (7)
C28—C27—H27	119.7	C34—C35—H35	119.5
C29—C28—C27	120.4 (6)	C36—C35—H35	119.5
C29—C28—H28	119.8	C17—C16—C15	120.0 (8)
C27—C28—H28	119.8	C17—C16—H16	120.0
C37—C32—C33	116.8 (6)	C15—C16—H16	120.0
C37—C32—P2	119.0 (5)	O4—C4—Fe2	176.2 (6)
C33—C32—P2	124.2 (5)		
			
C4—Fe2—Fe1—C3	−102.4 (3)	S1—Fe2—P2—C32	−140.4 (2)
P1—Fe2—Fe1—C3	118.4 (2)	S2—Fe2—P2—C32	−53.6 (3)
P2—Fe2—Fe1—C3	−7.8 (2)	Fe1—Fe2—P2—C32	−84.5 (2)
S1—Fe2—Fe1—C3	79.0 (2)	C4—Fe2—P2—C26	150.6 (3)
S2—Fe2—Fe1—C3	−174.3 (2)	P1—Fe2—P2—C26	−118.9 (3)
C4—Fe2—Fe1—C1	128.3 (4)	S1—Fe2—P2—C26	−13.2 (3)
P1—Fe2—Fe1—C1	−10.9 (4)	S2—Fe2—P2—C26	73.7 (3)
P2—Fe2—Fe1—C1	−137.1 (4)	Fe1—Fe2—P2—C26	42.8 (3)
S1—Fe2—Fe1—C1	−50.3 (4)	C6—N1—C5—S1	−66.0 (5)
S2—Fe2—Fe1—C1	56.4 (4)	C7—N1—C5—S1	169.8 (4)
C4—Fe2—Fe1—C2	−8.1 (3)	Fe1—S1—C5—N1	67.2 (4)
P1—Fe2—Fe1—C2	−147.3 (3)	Fe2—S1—C5—N1	−7.9 (5)
P2—Fe2—Fe1—C2	86.5 (2)	C6—N1—C7—C8	49.3 (7)
S1—Fe2—Fe1—C2	173.3 (2)	C5—N1—C7—C8	172.5 (5)
S2—Fe2—Fe1—C2	−80.0 (2)	C6—N1—C7—C11	170.6 (5)
C4—Fe2—Fe1—S1	178.6 (2)	C5—N1—C7—C11	−66.2 (6)
P1—Fe2—Fe1—S1	39.43 (14)	C12—P2—C26—C31	104.1 (6)
P2—Fe2—Fe1—S1	−86.77 (9)	C32—P2—C26—C31	−4.5 (6)
S2—Fe2—Fe1—S1	106.72 (9)	Fe2—P2—C26—C31	−135.7 (5)
C4—Fe2—Fe1—S2	71.9 (2)	C12—P2—C26—C27	−73.5 (6)
P1—Fe2—Fe1—S2	−67.29 (15)	C32—P2—C26—C27	177.9 (5)
P2—Fe2—Fe1—S2	166.50 (7)	Fe2—P2—C26—C27	46.7 (6)
S1—Fe2—Fe1—S2	−106.72 (9)	C22—C21—C20—C25	2.0 (9)
C3—Fe1—S2—C6	125.4 (6)	C22—C21—C20—P1	−174.9 (5)
C1—Fe1—S2—C6	−43.1 (3)	C14—P1—C20—C21	−166.1 (5)
C2—Fe1—S2—C6	−145.5 (3)	C13—P1—C20—C21	87.9 (5)
S1—Fe1—S2—C6	57.5 (2)	Fe2—P1—C20—C21	−28.7 (6)
Fe2—Fe1—S2—C6	110.2 (2)	C14—P1—C20—C25	17.0 (6)
C3—Fe1—S2—Fe2	15.2 (5)	C13—P1—C20—C25	−88.9 (5)
C1—Fe1—S2—Fe2	−153.3 (2)	Fe2—P1—C20—C25	154.4 (4)
C2—Fe1—S2—Fe2	104.3 (2)	N1—C7—C11—C10	−134.1 (6)
S1—Fe1—S2—Fe2	−52.71 (5)	C8—C7—C11—C10	−8.2 (7)
C4—Fe2—S2—C6	143.8 (3)	C31—C26—C27—C28	2.4 (9)
P1—Fe2—S2—C6	55.1 (2)	P2—C26—C27—C28	−179.9 (5)
P2—Fe2—S2—C6	−138.6 (3)	C30—C29—C28—C27	−0.5 (10)
S1—Fe2—S2—C6	−49.1 (2)	C26—C27—C28—C29	−0.5 (10)
Fe1—Fe2—S2—C6	−101.8 (2)	C12—P2—C32—C37	170.4 (5)
C4—Fe2—S2—Fe1	−114.4 (2)	C26—P2—C32—C37	−85.1 (5)
P1—Fe2—S2—Fe1	156.91 (6)	Fe2—P2—C32—C37	54.2 (5)
P2—Fe2—S2—Fe1	−36.82 (17)	C12—P2—C32—C33	−6.4 (6)
S1—Fe2—S2—Fe1	52.71 (7)	C26—P2—C32—C33	98.0 (6)
C4—Fe2—P1—C20	−178.1 (3)	Fe2—P2—C32—C33	−122.7 (5)
P2—Fe2—P1—C20	92.4 (2)	C21—C20—C25—C24	−1.0 (10)
S1—Fe2—P1—C20	−3.8 (2)	P1—C20—C25—C24	175.9 (5)
S2—Fe2—P1—C20	−92.7 (2)	C5—N1—C6—S2	70.1 (5)
Fe1—Fe2—P1—C20	−36.8 (3)	C7—N1—C6—S2	−166.8 (4)
C4—Fe2—P1—C14	−47.7 (3)	Fe1—S2—C6—N1	−75.7 (4)
P2—Fe2—P1—C14	−137.2 (2)	Fe2—S2—C6—N1	−0.9 (5)
S1—Fe2—P1—C14	126.7 (2)	C20—P1—C14—C15	89.2 (6)
S2—Fe2—P1—C14	37.7 (2)	C13—P1—C14—C15	−166.9 (6)
Fe1—Fe2—P1—C14	93.7 (3)	Fe2—P1—C14—C15	−50.2 (7)
C4—Fe2—P1—C13	67.7 (3)	C20—P1—C14—C19	−88.1 (6)
P2—Fe2—P1—C13	−21.8 (2)	C13—P1—C14—C19	15.8 (6)
S1—Fe2—P1—C13	−118.0 (2)	Fe2—P1—C14—C19	132.5 (5)
S2—Fe2—P1—C13	153.1 (2)	C20—C21—C22—C23	−2.1 (9)
Fe1—Fe2—P1—C13	−150.9 (2)	C21—C22—C23—C24	1.0 (10)
C3—Fe1—S1—C5	145.9 (3)	N1—C7—C8—C9	154.6 (6)
C1—Fe1—S1—C5	48.6 (3)	C11—C7—C8—C9	29.9 (7)
C2—Fe1—S1—C5	−123.0 (5)	C32—P2—C12—C13	−89.4 (5)
S2—Fe1—S1—C5	−54.2 (2)	C26—P2—C12—C13	164.5 (4)
Fe2—Fe1—S1—C5	−107.0 (2)	Fe2—P2—C12—C13	30.8 (5)
C3—Fe1—S1—Fe2	−107.1 (2)	C15—C14—C19—C18	−1.9 (10)
C1—Fe1—S1—Fe2	155.6 (2)	P1—C14—C19—C18	175.4 (5)
C2—Fe1—S1—Fe2	−15.9 (5)	C37—C32—C33—C34	1.3 (10)
S2—Fe1—S1—Fe2	52.84 (6)	P2—C32—C33—C34	178.2 (6)
C4—Fe2—S1—C5	100.3 (7)	C14—C19—C18—C17	0.5 (11)
P1—Fe2—S1—C5	−60.3 (2)	C27—C26—C31—C30	−3.3 (10)
P2—Fe2—S1—C5	−150.2 (2)	P2—C26—C31—C30	179.1 (5)
S2—Fe2—S1—C5	52.0 (2)	C33—C32—C37—C36	−1.6 (10)
Fe1—Fe2—S1—C5	104.7 (2)	P2—C32—C37—C36	−178.7 (5)
C4—Fe2—S1—Fe1	−4.5 (7)	P2—C12—C13—P1	−49.0 (5)
P1—Fe2—S1—Fe1	−165.07 (6)	C20—P1—C13—C12	−83.2 (5)
P2—Fe2—S1—Fe1	105.12 (8)	C14—P1—C13—C12	171.8 (4)
S2—Fe2—S1—Fe1	−52.76 (6)	Fe2—P1—C13—C12	45.8 (4)
C4—Fe2—P2—C12	−92.2 (3)	C19—C14—C15—C16	2.8 (13)
P1—Fe2—P2—C12	−1.6 (2)	P1—C14—C15—C16	−174.6 (8)
S1—Fe2—P2—C12	104.1 (2)	C28—C29—C30—C31	−0.5 (11)
S2—Fe2—P2—C12	−169.1 (2)	C26—C31—C30—C29	2.4 (11)
Fe1—Fe2—P2—C12	160.0 (2)	C7—C11—C10—C9	−17.5 (8)
C4—Fe2—P2—C32	23.3 (3)	C11—C10—C9—C8	36.4 (9)
P1—Fe2—P2—C32	113.8 (2)	C7—C8—C9—C10	−40.6 (8)

### Hydrogen-bond geometry (Å, °)

**Table:**

*D*—H···*A*	*D*—H	H···*A*	*D*···*A*	*D*—H···*A*
C21—H21···S1	0.93	2.77	3.490 (7)	135
C27—H27···S1	0.93	2.84	3.416 (7)	122
